# Superior Vena Cava Thrombosis Presenting As Massive Chylothorax: A Report of a Rare Case

**DOI:** 10.7759/cureus.86138

**Published:** 2025-06-16

**Authors:** Mohamed Zuhail K Peediyakkal, Nabil S Mahmood, Ashib Thurakkal, Ezzeddin Ibrahim, Nevin Kannappilly, Sunil H Koya, Saifil Sidhique, Bara M Al-Qudah, Abdulqadir J Nashwan

**Affiliations:** 1 Critical Care Medicine, Hamad Medical Corporation, Doha, QAT; 2 Radiology, Hamad Medical Corporation, Doha, QAT; 3 Nursing and Midwifery Research, Hamad Medical Corporation, Doha, QAT

**Keywords:** central venous thrombosis, chylothorax, end stage renal disease, hemodialysis, indwelling catheter, pleural effusion, superior vena cava syndrome, thoracic duct obstruction, uncommon complications, vascular access complications

## Abstract

Chylothorax is an uncommon condition resulting from the disruption or obstruction of the thoracic duct or its tributaries, leading to the accumulation of chyle in the pleural cavity. It can arise from both traumatic and non-traumatic causes. Non-traumatic etiologies include malignancies involving the mediastinum, followed by infections such as tuberculosis, connective tissue disorders (e.g., sarcoidosis, systemic lupus erythematosus), and, less frequently, radiation exposure and certain drugs. Although thoracic central venous thrombosis (CVT) is a recognized etiology, it remains a rare cause of chylothorax in adults. We report a rare case of massive chylothorax secondary to chronic CVT associated with a long-term indwelling central venous catheter in a patient undergoing hemodialysis for end-stage renal disease. The diagnosis of chylothorax was confirmed through pleural fluid analysis, which revealed characteristic findings, while the underlying cause was identified using targeted imaging modalities. This case highlights chylothorax's diagnostic complexity and clinical challenges in central venous obstruction. It also emphasizes the importance of maintaining a high index of suspicion in patients with relevant risk factors. A brief review of the underlying pathophysiology and available treatment approaches is also provided to aid clinicians in managing similar presentations.

## Introduction

Chylothorax is characterized by the accumulation of chyle, a triglyceride-rich, milky lymphatic fluid, in the pleural cavity [1]. Chyle is formed in the small intestine during digestion and absorbed into the lymphatic system, ultimately draining via the thoracic duct into the left subclavian or internal jugular vein. The thoracic duct originates in the abdomen and ascends through the thorax, making it susceptible to injury or compression along its course.

The etiology of chylothorax may be traumatic (e.g., thoracic surgery) or non-traumatic, including malignancies, infections such as tuberculosis, and connective tissue diseases [1,2]. Clinically, patients present with dyspnea, chest discomfort, and signs of pleural effusion. Diagnosis is typically established through pleural fluid analysis, revealing a milky appearance with elevated triglycerides and lymphocytic predominance. Treatment typically involves drainage of the chyle, dietary modifications, and in some cases, surgical intervention. It carries a high risk of mortality and morbidity if not treated promptly [1,2].

Central venous thrombosis (CVT), particularly involving the superior vena cava (SVC), is a rare and under-recognized cause of chylothorax in adults [3-5]. Most reported cases are linked to long-term indwelling central venous catheters. The mechanism involves elevated venous pressure proximal to the thrombosis, which disrupts lymphatic drainage by increasing thoracic duct backpressure, leading to chyle leakage into the pleural space. Diagnosing CVT-related chylothorax poses a challenge due to its insidious onset, overlapping clinical features with other pleural effusions, and the requirement for advanced imaging to confirm the vascular obstruction and exclude thoracic duct injury.

This report presents a rare case of massive chylothorax secondary to chronic SVC thrombosis in a patient undergoing hemodialysis through a long-term central venous catheter. It highlights the diagnostic complexities and clinical decision-making involved in managing this condition. Our case adds to the limited body of literature by emphasizing the importance of timely imaging, multidisciplinary collaboration, and vigilance in patients with prolonged catheter use. By doing so, we aim to raise awareness of this uncommon but clinically significant complication and enhance clinicians’ ability to recognize and address it early.

## Case presentation

A 51-year-old woman with a history of diabetes mellitus, hypertension, and end-stage renal disease (ESRD) on hemodialysis presented with a three-day history of progressive shortness of breath. She had recently been discharged following treatment for a tibial fracture. Hemodialysis was administered through a right internal jugular tunneled catheter that had been in place for six years. Her vascular history included a brachio-axillary graft thrombectomy performed six years ago for a thrombosed arteriovenous fistula, followed by another thrombectomy and bypass procedure in 2018.

On arrival at the emergency department, the patient was tachypneic and required supplemental oxygen. A computed tomography (CT) scan of the chest revealed a massive right-sided pleural effusion with associated lung collapse. In addition, the scan identified pulmonary embolism involving the segmental and subsegmental branches bilaterally. A 12F pigtail catheter was inserted to drain the pleural fluid, and therapeutic anticoagulation was initiated for the pulmonary embolism. The development of pulmonary embolism was likely multifactorial, with contributing factors including her recent immobilization following a tibial fracture, presence of a long-term central venous catheter, and underlying prothrombotic risk due to ESRD. Echocardiography showed normal cardiac function with no pericardial effusion.

The pleural fluid was milky in appearance and met the criteria for an exudate. Laboratory analysis demonstrated a high pleural fluid protein level (47.1 g/L), elevated triglycerides (15.8 mmol/L), and a cholesterol level of 2.4 mmol/L. The pleural fluid-to-serum protein ratio was 0.70, and the lactate dehydrogenase (LDH) ratio was 1.08. Lymphocytic predominance was noted at 65% (Table 1). Importantly, bacteriological cultures and Gram stain of the pleural fluid consistently showed no growth, ruling out an active superinfection of the chylothorax.

**Table 1:** Pleural fluid analysis LDH: lactate dehydrogenase, g/L: grams per liter, IU/L: international units per liter, mmol/L: millimoles per liter

Parameter	Result	Reference Range
Appearance	Milky	Clear to straw-colored
Colour	Yellow	Pale yellow
White blood cells (WBC)	688/mm^3^	<1,000/mm^3^
Red blood cells (RBC)	4,125/mm^3^	Variable (usually low)
Neutrophils	30%	Typically <25% in chylothorax
Lymphocytes	65%	>50% in chylothorax
Glucose	15.4 mmol/L	3.3-5.5 mmol/L
Protein	47.1 g/L	<30 g/L (transudate); >30 g/L (exudate)
LDH	265 IU/L	<200 IU/L (transudate); >200 IU/L (exudate)
Cholesterol	2.4 mmol/L	<5.2 mmol/L
Triglycerides	15.8 mmol/L	>1.24 mmol/L suggests chylothorax

Due to blockage of the initial drain, the pigtail catheter was replaced with a 24F chest tube. Despite this, the patient continued to have high-output chylous drainage. A repeat chest CT showed no reduction in the chylothorax and identified chronic thrombosis of the SVC with the development of collateral circulation. She subsequently developed left subclavian vein thrombosis following insertion of a peripherally inserted central catheter (PICC), which was necessitated by difficult intravenous access.

**Figure 1:**
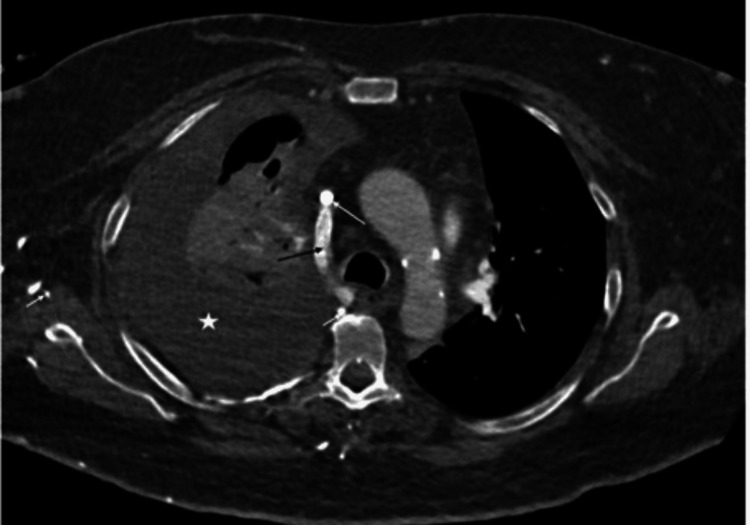
Axial post-contrast CT study of the thorax Axial post-contrast CT image of the thorax after intravenous injection into the right subclavian vein above, at, and below the level of the azygous vein-superior vena cava (SVC) confluence shows massive right-sided pleural effusion in keeping with chylothorax (white asterisk). A right-sided central venous catheter is noted with non-visualization of an enhancing SVC, suggesting chronic thrombosis (long white arrow).

Given the persistent chyle leak, a CT scan of the abdomen was performed to evaluate for intra-abdominal malignancy or lymphadenopathy. The scan showed only minimal pelvic free fluid and signs of colitis. The patient was initiated on a low-fat parenteral nutrition regimen to manage the ongoing chylous output (Figure 1).

**Figure 2:**
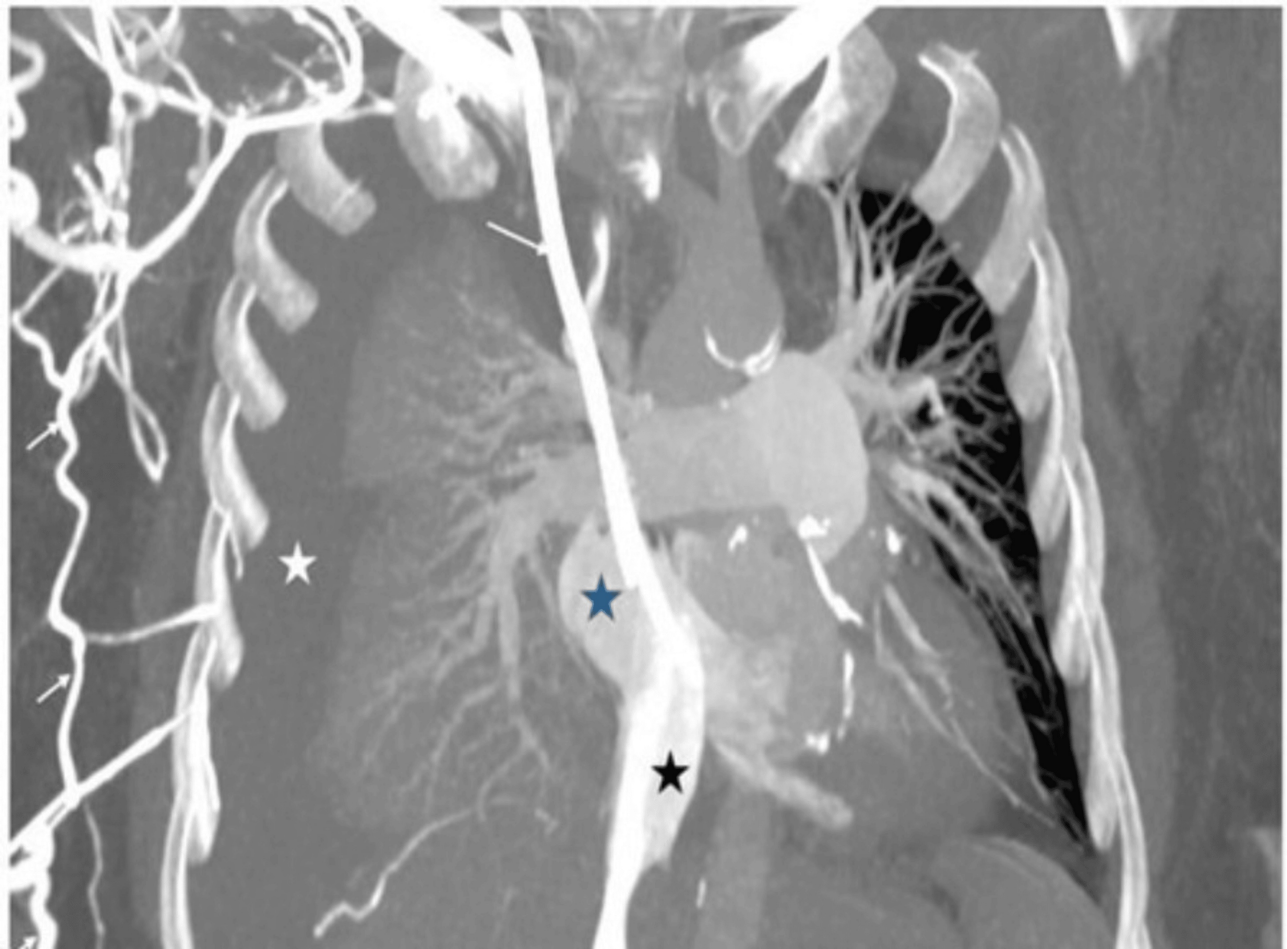
Post-contrast MIP image Post-contrast maximum intensity projection (MIP) image in the coronal plane shows massive right-sided pleural effusion in keeping with chylothorax (white asterisk). A right-sided central venous catheter is noted with non-visualization of an enhancing SVC, suggesting chronic thrombosis. Numerous venous collaterals are noted (white arrow), bypassing the venous drainage from the right subclavian vein into the azygous vein and subsequently reforming the terminal segment of the SVC. Normal contrast opacification and caliber of the IVC (black asterisk) draining into the right atrium (blue asterisk) is noted. SVC: superior vena cava; IVC: inferior vena cava

**Figure 3:**
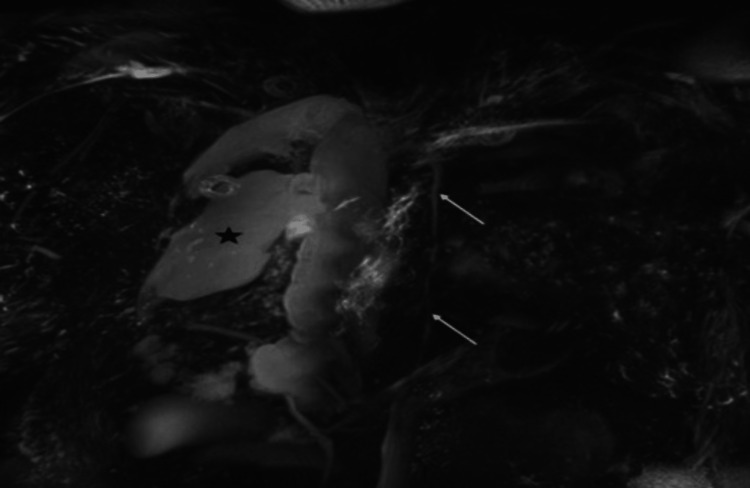
Coronal oblique MIP image from non-contrast MR lymphography Maximum intensity projection (MIP) coronal oblique image from a non-contrast MR lymphogram study shows a thoracic duct of normal caliber with no suggestion of injury (long white arrows). Chylothorax is seen on the right side (black asterisk).

Finally, a magnetic resonance lymphangiogram was performed to rule out thoracic duct injury. It demonstrated pleural effusion, SVC thrombosis, and right distal subclavian vein thrombosis (Figures 2-3).

The thoracic duct was found to be normal. The treatment plan in this case included a multidisciplinary approach, involving critical care, vascular surgery, and interventional radiology teams. Systemic anticoagulation was initiated upon the diagnosis of pulmonary embolism and subsequently continued to address the identified CVT. Catheter-related thrombosis was suspected based on imaging and clinical context, and removal of the long-term right internal jugular catheter was considered. However, the patient became critically ill due to sepsis before catheter extraction could be safely attempted. Endovascular intervention, including angioplasty and stent placement, was also under consideration for refractory chylothorax, but could not be pursued due to her rapid clinical deterioration. The primary suspected focus of infection was a catheter-related bloodstream infection, given her multiple indwelling central venous lines. Blood cultures drawn at this time were negative for any microbial etiology. Despite aggressive antibiotic therapy, supportive measures, including vasopressor support and mechanical ventilation, she unfortunately succumbed to her illness.

## Discussion

Chylothorax is the accumulation of chyle, a milky fluid composed of lymph and emulsified fats from the gastrointestinal tract, within the pleural space [5]. The condition may arise from a range of etiologies that are broadly classified as traumatic or non-traumatic. Traumatic or iatrogenic causes include procedures such as central venous catheter placement, pacemaker insertion, blunt chest trauma, and thoracic surgeries [6,7].

Non-traumatic causes are further categorized into malignant and non-malignant conditions. Malignant causes commonly include mediastinal tumors, lung cancer, metastatic mediastinal lesions, and lymphomas. Non-malignant etiologies encompass SVC syndrome [8], tuberculosis [9], thoracic irradiation [10], thoracic central venous thrombosis (TCVO), retrosternal goiter, mediastinal granulomatous diseases [11], and connective tissue disorders.

The thoracic duct originates from the cisterna chyli in the abdomen and ascends through the aortic hiatus into the thoracic cavity. It runs along the right side of the midline between the aorta and azygos vein and lies posterior to the esophagus [12,13]. Ultimately, it drains lymph into the junction of the left internal jugular and subclavian veins. Understanding thoracic duct anatomy is essential in managing chylothorax. Injuries or obstructions above the T4-T6 level typically cause left-sided chylothorax, whereas those below this level result in right-sided effusions [14].

TCVO is a recognized complication in patients with ESRD on hemodialysis. TCVO may result from thrombus formation or long-term use of intravascular devices such as tunneled dialysis catheters [15]. These catheters can lead to luminal narrowing due to endothelial injury and intimal hyperplasia, predisposing the vessel to complete occlusion [16].

SVC thrombosis, a form of TCVO, has a range of underlying causes, including indwelling catheters, malignancies, and prothrombotic conditions. Management options vary from conservative treatment with anticoagulation to interventional procedures such as thrombolysis and stent placement. Decisions must be individualized based on patient comorbidities and underlying pathology.

SVC thrombosis can lead to venous congestion of the upper body, manifesting as SVC syndrome. SVC thrombosis has also been implicated as a rare cause of chylothorax [17,18]. The proposed mechanism involves increased venous pressure distal to the site of thrombosis, which is transmitted retrogradely to the thoracic duct. This elevated pressure disrupts lymphatic drainage and causes chyle to leak into the pleural cavity [19].

Effective management of chylothorax begins with addressing the underlying cause. Initial steps typically include pleural drainage and nutritional modifications such as a low-fat diet enriched with medium-chain triglycerides or parenteral nutrition [20]. When conservative measures fail, more invasive strategies, such as pleurodesis, thoracic duct ligation, or embolization, should be considered [21]. Treatment options for SVC thrombosis cases include systemic anticoagulation and endovascular procedures such as balloon angioplasty and stent placement, particularly in patients with recurrent symptoms or refractory chylothorax [21].

This case underscores several critical learning points for clinicians. Firstly, it highlights the importance of maintaining a high index of suspicion for CVT as a rare but significant cause of chylothorax, particularly in patients with a history of long-term indwelling central venous catheters, such as those undergoing hemodialysis. While pleural fluid analysis confirms chylothorax, targeted imaging modalities are crucial for identifying the underlying cause. Our case demonstrates how repeated chest CT and magnetic resonance lymphangiography were instrumental in diagnosing chronic SVC thrombosis and excluding thoracic duct injury, which is vital for guiding appropriate management. Finally, the rapid clinical deterioration due to sepsis emphasizes the vulnerabilities of patients with long-standing central venous access and comorbidities, underscoring the urgency of definitive diagnosis and intervention in these complex cases.

## Conclusions

In our patient, chylothorax was directly diagnosed through characteristic pleural fluid analysis, while SVC thrombosis was unequivocally confirmed using contrast-enhanced CT and MR lymphangiography, which demonstrated the thrombus and associated collateral circulation. The causative link between SVC thrombosis and chylothorax was established based on the direct evidence of venous obstruction, the well-known pathophysiological relationship between central venous obstruction and impaired lymphatic drainage, the presence of a major risk factor (long-term central venous catheter), and the exclusion of alternative causes such as malignancy or thoracic duct injury - supported by a normal thoracic duct appearance on imaging. This case underscores the value of a systematic diagnostic approach, especially when conservative management fails or the clinical scenario raises suspicion for less common etiologies. Advanced imaging techniques play a vital role in identifying rare causes and should be employed early to avoid diagnostic delays and prevent morbidity.

Clinicians should maintain a high index of suspicion for central venous access complications in patients with long-term devices, particularly when faced with atypical presentations like chylothorax. This case illustrates the diagnostic and therapeutic complexity associated with central venous obstruction and reinforces the need for timely recognition of early warning signs. Prompt identification of the underlying cause allows for tailored therapeutic interventions, such as anticoagulation, stenting, or surgical bypass, which are essential for achieving resolution in complex cases of chylothorax. Early intervention not only facilitates recovery but also reduces the risk of recurrence and long-term complications.
